# Development of a Nanostructured Platform for Identifying HER2-Heterogeneity of Breast Cancer Cells by Surface-Enhanced Raman Scattering

**DOI:** 10.3390/nano8070549

**Published:** 2018-07-20

**Authors:** Alexandro Téllez-Plancarte, Emmanuel Haro-Poniatowski, Michel Picquart, José Guadalupe Morales-Méndez, Carlos Lara-Cruz, Javier Esteban Jiménez-Salazar, Pablo Damián-Matsumura, Luis Escobar-Alarcón, Nikola Batina

**Affiliations:** 1 Departamento de Química, Universidad Autónoma Metropolitana Iztapalapa, Av. San Rafael Atlixco No. 186, Col. Vicentina, C.P., Ciudad de México 09340, Mexico; alextellez9@xanum.uam.mx; 2 Departamento de Física, Universidad Autónoma Metropolitana Iztapalapa, Av. San Rafael Atlixco No. 186, Col. Vicentina, C.P., Ciudad de México 09340, Mexico; haro@xanum.uam.mx (E.H.-P.); mp@xanum.uam.mx (M.P.); jose_gmm@hotmail.com (J.G.M.-M.); 3 Departamento de Biología de la Reproducción, Universidad Autónoma Metropolitana Iztapalapa, Av. San Rafael Atlixco No. 186, Col. Vicentina, C.P., Ciudad de México 09340, Mexico; carritro@gmail.com (C.L.-C.); bioquimicajejs@hotmail.com (J.E.J.-S.); pgdm@xanum.uam.mx (P.D.-M.); 4 Departamento de Física, Instituto Nacional de Investigaciones Nucleares, Carretera México-Toluca S/N, C.P., La Marquesa Ocoyoacac 52750, Mexico; luis.escobar@inin.gob.mx

**Keywords:** atomic force microscopy, surface-enhanced Raman scattering, HER2, breast cancer cells

## Abstract

Biosensor technology has great potential for the detection of cancer through tumor-associated molecular biomarkers. In this work, we describe the immobilization of the recombinant humanized anti-HER2 monoclonal antibody (trastuzumab) on a silver nanostructured plate made by pulsed laser deposition (PLD), over a thin film of Au(111). Immobilization was performed via 4-mercapto benzoic acid self-assembled monolayers (4-MBA SAMs) that were activated with coupling reagents. A combination of immunofluorescence images and z-stack analysis by confocal laser scanning microscopy (CLSM) allowed us to detect HER2 presence and distribution in the cell membranes. Four different HER2-expressing breast cancer cell lines (SKBR3 +++, MCF-7 +/−, T47D +/−, MDA-MB-231 −) were incubated during 24 h on functionalized silver nanostructured plates (FSNP) and also on Au(111) thin films. The cells were fixed by means of an ethanol dehydration train, then characterized by atomic force microscopy (AFM) and surface-enhanced Raman scattering (SERS). SERS results showed the same tendency as CLSM findings (SKBR3 > MCF-7 > T47D > MDA-MB-231), especially when the Raman peak associated with phenylalanine amino acid (1002 cm^−1^) was monitored. Given the high selectivity and high sensitivity of SERS with a functionalized silver nanostructured plate (FSNP), we propose this method for identifying the presence of HER2 and consequently, of breast cancer cells.

## 1. Introduction

Breast cancer is the second cancer cause of death in the United States and it is estimated that 1.7 million new cancer cases will be diagnosed and 609,640 Americans will die of cancer in 2018 [1]. In Mexico, breast cancer is the fifth most common cause of death (n = 4496 average annual deaths from 2000–2013) and the leading cause of cancer mortality in Mexican women [2]. Tumor-associated antigens have been used as molecular biomarkers for cancer diagnosis; these biological molecules can be detected in or on tumor cells, blood, serum, urine, or cerebrospinal fluids, which are overexpressed due to cell cancer and growth [3].

HER2 is a member of the human epidermal growth factor receptor family, which regulates cell growth and differentiation and is also involved in some human cancers, such as breast and colon cancer [4,5]. The expression of HER2 proto-oncogene in normal breast epithelial cells that gives rise to a 185 kDa trans-membrane glycoprotein and its overexpression, found in 20%–30% of human breast cancers, correlates with more aggressive tumors and a poorer prognosis [6,7]. HER2 overexpression is caused by amplification of the *c-erbB-2* gene, which results in 1 to 2 million receptors per cell, compared to 20,000–50,000 receptors in normal breast epithelial cells [8]. Trastuzumab is a recombinant humanized anti-HER2 monoclonal antibody that selectively binds with high affinity to HER2 extracellular domain, inhibits cell proliferation, attacks HER2-dependent tumors and blocks shedding of HER2 extracellular domain [9,10]. HER2 detection has been used in different biosensors for breast cancer diagnosis and prognosis [11,12].

The surface plasmon resonance (SPR) refers to the collective oscillations of conduction electrons in metal nanostructures and this effect has been used in chemosensors and biosensors. SPR occurs in two different forms: localized surface plasmon resonance (LSPR) and surface plasmon polaritons (SPPs). The LSPR concentrates the incident electromagnetic field around the nanostructured films and this local electromagnetic field can influence processes, such as: plasmon enhanced fluorescence (PEF), surface-enhanced Raman scattering (SERS) and surface-enhanced infrared absorption spectroscopy (SEIRAS) and its associated evanescent electromagnetic field that extends to the surrounding medium around 30 nm of distance. In contrast, the SPPs concentrate the incident electromagnetic field around continuous or flat films and their evanescent electromagnetic field decays approximately to 200 nm of distance [13].

Surface-enhanced Raman scattering (SERS) turns the weak inelastic scattering effect of photons on vibrational quantum states into a structurally sensitive single-molecule and nanoscale probe [14]. SERS ‘hotspots’ are located at the junctions of nanoaggregates, where the local electromagnetic field amplitude is increased by plasmonic field confinement, enabling Raman amplifications up to 10^10^. Furthermore, the existence of the chemical effect due to chemisorption of molecules on surfaces enables Raman intensity enhancements up to 10^4^, taking into account that, both effects (electromagnetic and chemical) can produce an enhancement of the order of 10^14^ [15,16]. SERS has been used as a technique for biomarker biosensing in yeast, *H. pylori*, *E. coli* and hemoglobin A1c the authors propose a new hyper-spectral imaging system in order to resolve the intrinsic spatial inhomogeneity of SERS spectra [17]. Some of the different merits of SERS are the multiplicity of analyzed molecules against the fluorescence technique, as well as its sensitivity and robustness against laser radiation due to energy transfer from excited molecules to metal surfaces.

New methods for cancer detection, such as biosensors and more reliable molecular biomarkers, are needed for attaining the challenge of an early detection of the disease. A biosensor is an analytical device with a bioreceptor attached on its surface, which, when interacting with the analyte molecule, carries out a biochemical reaction or a specific union and by means of a signal transducer the resulting (bio)chemical signal is converted into an electric one. The intensity of the generated signal is directly proportional to the analyte concentration [18]. One type of biosensors is the immunosensor, which relies on the ability of an antibody to form a stable complex with its corresponding antigen [19].

Increased Raman peaks that correspond to phenylalanine (Phe) vibrational modes have been found in various cancer types, such as prostate, lung, breast, oral and esophageal tissue samples. In this regard, different researches were reported in the scientific literature. Li et al. [20] observed a considerable increase in Raman peaks at 1217 cm^−1^ (C-C_6_H_5_ bending mode of phenylalanine and tryptophan) and 1586 cm^−1^ (C=C bending mode of Phe, acetoacetate and riboflavin) in serum of prostate cancer patients when analyzed by SERS. Huang et al. [21] showed higher Raman signals for nucleic acids, tryptophan (Trp) and Phe in lung malignant tissue when analyzed by Raman spectroscopy. Zhu et al. [22] found Raman bands at 1004 and 1030 cm^−1^ (assigned to the symmetric ring breathing mode of Phe) remarkably enhanced in human breast cancer cells when analyzed by SERS. Girish et al. [23] reported intense SERS Raman peaks due especially to aromatic amino acids (Phe, Trp and tyrosine [Tyr]) in oral malignant tissue. Rekha et al. [24] have found marked differences in the Raman spectra of blood plasma from oral cancer patients with respect to healthy subjects, in particular, for the Raman peaks related to Phe and Trp, among others. González et al. [25] observed that Phe band intensity at 1002 cm^−1^ depends on the stage of breast cancer and finally, Feng et al. [26] found increased Raman signals at 1211 cm^−1^ (Phe) and 1583 cm^−1^ (Phe, riboflavin) in esophageal malignant tissue when analyzed by SERS.

In addition to the aforementioned examples, Chaturvedi et al. [27] monitored the biomolecular changes associated with the transformation of a normal cell into an invasive breast cancer cell using Raman microspectroscopy and showed the suitability of this technique as non-invasive and label-free, having the potential to probe changes in the biomolecular composition of living cells as well as fixed cells.

All these data demonstrate that Raman spectroscopy has potential for cancer sample diagnostics and reduces times, cost and traumas of surgical operations. However, the lack of high sensitivity and Raman peak definition, as well as interpretation, are still problems that must be addressed.

In the present work, we designed a FSNP that is able to recognize under pseudo-physiological conditions the HER2 extracellular domain in four breast cancer cell lines with differential expression of surface HER2 and such molecular recognition was detected by SERS. Although we are not the first to point out an increase in Raman intensity of the vibrational modes associated with phenylalanine, to the best of our knowledge, we are the first to point out that phenylalanine Raman peak could be used as a label associated with cancer cells and monitored by SERS through a FSNP, for their identification.

## 2. Experimental Section

### 2.1. Materials

MCF-7 (Luminal B), T47D (Luminal A) and MDA-MB-231 (HER2 negative) cell lines acquired from American Type Culture Collection (ATCC, Manassas, VA, USA), SKBR3 (overexpressing HER2) cell line was a gift from Dr. María Guadalupe Isabel Domínguez Gómez from INCan, Mexico City, Mexico. The following materials were used: Dulbecco’s modified eagle’s medium (DMEM), fetal bovine serum (FBS), RPMI medium, F12 medium, non-essential amino acids, l-glutamine, sodium pyruvate, nunclon 6-well multidish, Alexa Fluor 594 dye (secondary antibody) and Penicillin/Streptomycin (antibiotic/antifungal) were purchased from Thermo Fisher Scientific, Mexico City, Mexico.

*N*-(3-dimethylaminopropyl)-*N*′-ethylcarbodiimide hydrochloride (EDC·HCI) was purchased from Life Technologies, Mexico City, Mexico. Antibodies against HER2/ErbB2 (D8F12/4290P) were obtained from Cell Signaling Technology, Danvers, MA, USA. HER2/Neu MAb (sc-33684) and 4-mercapto benzoic acid (4-MBA) were purchased from Santa Cruz Biotechnology, Dallas, TX, USA. Ethanolamine hydrochloride, *N*-Hydroxysuccinimide (NHS) and 4′,6-diamidino-2-phenylindole (DAPI) were purchased from Sigma-Aldrich, Toluca, Mexico. Ultrapure water was used from a Milli-Q system (Millipore, Burlington, MA, USA), Herceptin (trastuzumab, generic name) was purchased from Roche-Mexico, 3.5 kDa MWCO dialysis membrane (Spectrum Labs, Rancho Dominguez, CA, USA) and other reagents were analytical grade. Au(111) plates for AFM and SERS measurements were obtained from Arrandee Co., Werther (Westfalen), Germany. 99.99% pure silver target (1.00 diameter × 0.259 thick) for PLD was obtained from Kurt J. Lesker Co., Jefferson Hills, PA, USA.

### 2.2. Methods

**Pulsed Laser Deposition (PLD).** Silver nanostructures on Au(111) substrates were prepared at room temperature by ablating a high purity silver target. Laser deposition was carried out in a vacuum chamber at a background pressure close to 2.7 × 10^−5^ torr, obtained with a turbomolecular pump. The third harmonic (λ = 355 nm) of a Q-switched Nd:YAG laser (Spectra Physics), delivering 10 mJ in pulses of 10 nanoseconds of duration, working at a repetition rate of 10 Hz, was used as energy source. The laser beam was focused on the silver target surface using a quartz lens of 50 cm of focal length, which produced a spot of 0.80 mm mean diameter and an average laser fluence close to 2.0 J/cm^2^. Au(111) plates described above were used as substrates for PLD, placed at a distance of 6.0 cm directly in front of the silver target and subsequently, 3000 laser pulses were used to produce silver nanostructures (45.0 ± 16.7 nm) on a surface of approximately 0.5 cm^2^.

**Atomic Force Microscopy (AFM).** All AFM images (substrates, cell plasma membranes and surface analysis of cell plasma membranes) were characterized at room temperature under atmospheric conditions, in the tapping mode with a NanoScope IIIa (Veeco Instruments Inc., Santa Barbara, CA, USA). Probes of antimony (n) doped silicon that had an average resonance frequency of 317–382 kHz with a spring constant of 20–80 N/nm were used at a scan rate of 0.4–0.7 Hz. The amplitude values of the cantilever remained constant at approximately 3000 mV and at 1.5 V set point. AFM images were manually obtained with a mean time of 45 min per sample; the acquisition modes were height and phase and their analyses were performed through the AFM NanoScope III software. Each sample (cell) was examined at five different places over the cell surface in two different series of samples.

**Raman and Surface-enhanced Raman scattering (SERS) measurements.** All Raman and SERS spectra were recorded at least three times in three different points of the sample, at room temperature with a Horiba Jobin-Yvon LabRam HR 800 micro-Raman system, equipped with an Olympus BX40 confocal microscope (Edison, NJ, USA) and a CCD detector (Edison, NJ, USA). Measurements were performed using an excitation wavelength of 532.1 nm, a 50× objective, at a power close to 7 mW on the sample and 100 accumulations of 10 s per spectrum to improve the signal-to-noise (S/N) ratio. The spectra were calibrated using the 521 cm^−1^ line of monocrystalline silicon. In order to take spatially similar Raman spectra, these were taken near the cell nuclei in all the cases.

**Breast cancer cell line cultures.** MCF-7 was seeded in Petri dishes containing phenol red-free DMEM supplemented with 2.5% (*v*/*v*) FBS. T47D and MDA-MB-231 were cultured with RPMI medium and 2.5% (*v*/*v*) FBS. SKBR3 was cultured with DMEM-F12 medium (3:1 *v*/*v*) and 10% (*v*/*v*) FBS. All culture media were supplemented with non-essential amino acids (100 mM), antibiotic/antifungal (Penicillin 100 U/mL/Streptomycin 100 mg/mL), l-glutamine (2 mM) and sodium pyruvate (100 mM) at 1% (*v*/*v*). Breast cancer cell lines were cultured in a humidified atmosphere (5% CO_2_ and 37 °C) until 100% confluence, removed by means of trypsin-EDTA, centrifuged at 1500× *g* during 10 min and re-suspended in new culture medium.

**Trastuzumab antibody purification.** One mg of Herceptin was dissolved in 1 mL of 10 mM of PBS buffer at pH 8.3 and subsequently dialyzed overnight, using a dialysis membrane of 3.5 kDa MWCO against 1 L of the same buffer. The concentration of trastuzumab was calculated using the Beer-Lambert law, taking into account the following constants: ε_280nm_ = 2.25 × 10^5^ M^−1^cm^−1^, MW = 145.53 kDa and measuring its absorbance at 280 nm with a BioDrop spectrophotometer [28,29]. Dilutions of trastuzumab at 100 µgmL^−1^ were used for its immobilization on 4-MBA SAMs. Trastuzumab shows an isoelectric point at pH 8.8, so a pH at 0.5 units below its isoelectric point is optimum for amine coupling via NHS-ester intermediates [30].

**4-Mercapto Benzoic Self-Assembled Monolayers (4-MBA SAMs) preparation.** Fresh silver nanostructured substrates produced by PLD (3000 laser pulses of silver irradiated on an Au(111) thin film) were immersed for 15 s into an ethanol solution of 10 mM of 4-MBA, then cautiously rinsed in an acetic acid solution at pH 1.8 and gently dried [31]. Before use, the substrates were autoclaved.

**Functionalized Silver Nanostructured Plate (FSNP) Design.** Solutions of 0.4 M of EDC·HCI, 0.1 M of NHS and 1.0 M of ethanolamine hydrochloride (adjusted with HCI at pH 8.5) were prepared using water as solvent, separated in small aliquots, frozen at −20 °C and used until required [32]. 4-MBA SAMs were activated during 10 min with 15 µL of EDC/NHS (1:1 *v*/*v*) coupling reagents and afterwards, gently dried; 10 µL of trastuzumab at 100 µgmL^−1^ was deposited for 20 min and again gently dried; 15 µL of ethanolamine hydrochloride solution was deposited for 7 min for surface deactivation and finally FSNP was rinsed with PBS and immediately used.

**Confocal Laser Scanning Microscopy (CLSM).** The coverslips for immunocytochemistry were previously washed and autoclaved. In a 6-well multidish cell culture plate, 4 coverslips were placed for each cell line; approximately 150,000 cells were dropped on each coverslip and incubated for 24 h. After this time, the culture medium was withdrawn, coverslips were rinsed 3× with tempered PBS-Tween 20 at 1% (*v*/*v*), 1 mL of 4% neutral buffered formalin was added and left to stand for 20 min at room temperature.

Once again, coverslips were rinsed 3× with tempered PBS, primary anti-HER2 monoclonal antibody from mouse dissolved in PBS at 1:100 (*v*/*v*) was dropped on each coverslip and incubated during 1.5 h, then rinsed 3× with tempered PBS. One more time, coverslips were incubated with an aliquot of anti-anti-HER2 secondary antibody from mouse, bound to Alexa Fluor 594 fluorophore dissolved in PBS at 1:100 (*v*/*v*) that was dropped on each coverslip and incubated during 1.5 h. Finally, nuclei were counterstained with a solution of DAKO DAPI (2 ng/mL) that was placed on each coverslip for 10 min, then coverslips were sealed and refrigerated at 4 °C until use.

## 3. Results and Discussion

**Scheme 1:**
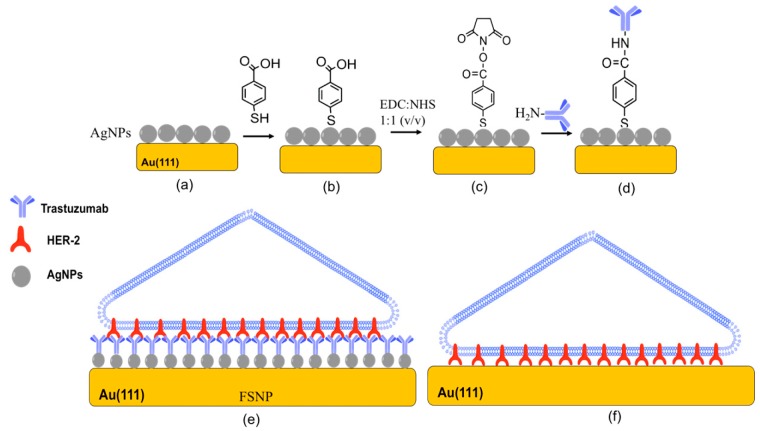
Schematic representation of (**a**) silver nanostructures deposited by pulsed laser detection (PLD) on Au(111) substrate; (**b**) formation of 4-mercapto benzoic acid self-assembled monolayers (4-MBA SAMs) on the silver nanostructured substrate; (**c**) formation of the reactive intermediate (NHS-ester) for amine coupling; (**d**) formation of a functionalized silver nanostructured plate (FSNP) with anti-HER2 (trastuzumab); (**e**) a breast cancer cell attached on a FSNP and (**f**) a breast cancer cell attached on a plain Au(111) substrate (control).

In order to find a method for simple and direct detection of breast cancer, we designed a platform for the identification of HER2 surface receptor on plasma membranes. *As was mentioned in the introduction, the overexpression of HER2 is found in 20%–30% of human breast cancers and it correlates with more aggressive tumors and a poorer prognosis [6]. See Scheme 1*.

**Figure 1:**
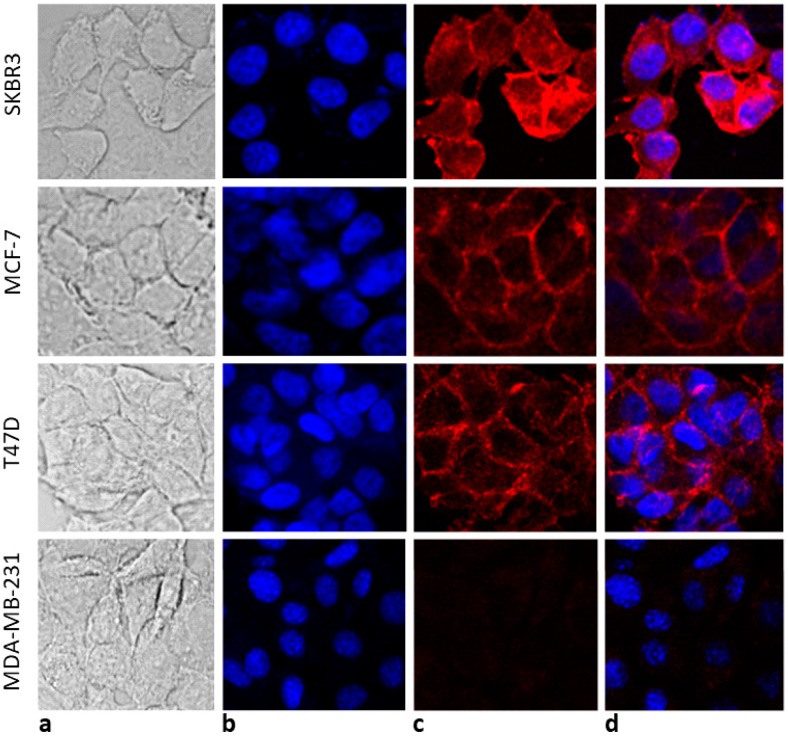
Images of immunofluorescence by confocal laser scanning microscopy (CLSM) of four different breast cancer cell lines: MCF-7, SKBR3, T47D and MDA-MB-231 (**a**) bright field; (**b**) staining of the cell nucleus by the fluorophore DAPI (blue); (**c**) indirect detection of HER2 by a primary anti-HER2 antibody and an anti-anti-HER2 secondary antibody bound to Alexa Fluor 594 fluorophore (red) and (**d**) cell localization of HER2 receptor by overlapping of images (DAPI + HER2). Magnification: 63×.

In the present work, we studied four cell lines: SKBR3, MCF-7, T47D and MDA-MB-231, with different HER2 expression levels (from high to low). Figure 1a–d shows the results of CLSM analysis revealing the presence and distribution of HER2 surface receptors in different breast cancer cell lines (red color due to Alexa Fluor 594 fluorophore), previously identified by immunofluorescence technique. The analysis of CLSM images from Figure 1d corroborated that the SKBR3 cell line exhibited high expression of HER2 surface receptor, being 80%–90% of the total cells analyzed on the surface of the membrane and the rest dispersed in the cytoplasm. In MCF-7 cells, HER2 surface receptor was on the membrane in 40%–60% of the total cells analyzed and could also be observed in the nucleus, especially in cells with higher expression; this effect was not observed in SKBR3 cells but its location in these cells was corroborated by the Z-stack analysis (not shown).

The expression of HER2 on the membrane surfaces of T47D cells was observed in less than 20% of the total cells analyzed, with less intensity than in the other two cell lines and preferably, in the cytoplasm and nucleus; and lastly, no HER2 expression was found in MDA-MB-231 cells. These results allow us to establish the differential expression of HER2 present on the cell membranes in the following way: SKBR3 > MCF-7 > T47D > MDA-MB-231.

In general, these results are in good agreement with the findings already documented in the scientific literature that SKBR3 is a HER2-overexpressing cell line [33], MCF-7 and T47D are low HER2-overexpressing cell lines [34] and MDA-MB-231 is considered triple negative (no expression of HER2, progesterone and estrogen receptors) [35]. Russo et al. reported that HER2 expressed at T47D is predominantly cytosolic [36]. Therefore, MCF-7 cell line presents more HER2 surface receptors than T47D cell line. It is worthwhile mentioning that for immuno-fluorescence investigations, the substrates have to be transparent (glass plates) due to the optical configuration. Furthermore, the glass plates have, in this case, a transparent polymer thin film that enables cell adhesion. Such substrates were not suitable for AFM and SERS studies.

In order to find a substrate with high affinity and selectivity for breast cancer cells, based on their HER2 expression, we designed a special functionalized silver nanostructured plate (FSNP) with an anti-HER2 layer on a flat Au(111) substrate. This surface is good for AFM analysis and the silver nanoparticle layer is essential to produce SERS, since it requires a nanostructured surface to enhance the Raman signal and, at the same time, improve the S/N ratio of the adsorbate. Note, glass substrates are significantly rougher than Au(111) surface, making difficult the analysis by AFM. Our functionalized silver nanostructured plate (FSNP) is highly defined (homogeneous) with AgNPs of a defined size that can be used as a reference. In the case of glass substrates, it is very difficult to obtain the same characteristics.

Search for the optimum FSNP was carried out as a large and detailed study (not shown), however here we present only the most relevant results. In short, we used PLD technique, with 3000 nanosecond laser pulses to deposit silver nanoparticles of the desired size onto the flat Au(111) substrate. According to Stamplecoskie et al., the silver nanoparticles of 50 nm diameter are favorable for SERS phenomena [37]. A great number of experiments was performed on different substrates by varying the number of laser pulses, from 500–3000, until finding the best experimental conditions that provided the highest signal intensity and the highest SERS effect, that is, the highest S/N ratio.

**Figure 2:**
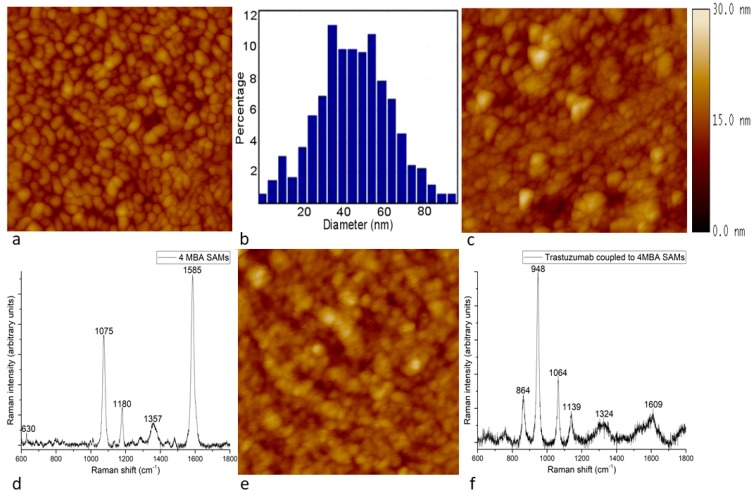
(**a**) Atomic force microscopy (AFM) image of a silver nanostructured substrate prepared by PLD at vacuum; (**b**) statistical analysis of the diameter of silver nanostructures; (**c**) AFM image of 2D morphology of 4-MBA SAMs formed on the silver nanostructured substrate; (**d**) Raman spectrum of 4-MBA SAMs formed on the silver nanostructured substrate; (**e**) AFM image of 2D morphology of FSNP with an anti-HER2 layer and (**f**) Raman spectrum of FSNP. AFM images are (1 µm × 1 µm) and share the same height scale (z-scale: 0–30 nm).

Our nanostructured films were characterized and optimized for SERS, using sodium dodecyl sulfate (SDS) as a test molecule (20 mM of SDS dissolved at 1:2 *v*/*v* chloroform-ethanol). Figure 2a shows an AFM image of the silver nanostructured film prepared by PLD on a flat Au(111) substrate. Figure 2b shows the statistical analysis performed by SPIP 3D Image Processing v6.2.4. (Image Metrology, Horsholm, Denmark) whose results were: density of 538 silver nanostructures/µm^2^ and 45.0 ± 16.7 nm of diameter. In the process of further modification of 4-MBA SAMs, a layer with NHS-esters was formed and trastuzumab was deposited on the nanostructured silver/gold substrate. For more details, see methodology section of this paper. Each step of modification was followed by a detailed characterization (AFM, SERS) of the morphology and optical properties. Figure 2c shows an AFM image of 4-MBA SAMs formed on the nanostructured silver/gold substrate in which some features of the nanostructured surface are clearly observed. Figure 2d exhibits the Raman spectrum of 4-mercapto benzoic acid self-assembled monolayers (4-MBA SAMs), which is characterized by Raman peaks at 630 cm^−1^ (C-S), 1075 cm^−1^ (CH in-plane bending), 1180 cm^−1^ (CH bending), 1357 cm^−1^ (COO^−^ stretching) and 1585 cm^−1^ (C=C stretching). Figure 2e shows an AFM image of trastuzumab oriented-immobilized on 4-MBA SAMs to form a FSNP; again, the nanostructured characteristics of the substrate are observed. Figure 2f shows the Raman spectrum of FSNP, which is characterized by Raman peaks at 864 cm^−1^ (Tyr), 948 cm^−1^ (N-C_α_-C), 1064 cm^−1^ (C-C or alkyl C-N), 1139 cm^−1^ (C-C or alkyl C-N), 1324 cm^−1^ (Trp, C_α_-H) and 1609 cm^−1^ (Tyr, Trp, Phe). All Raman peaks were assigned according to the literature [38,39]. The disappearance of the 4-MBA Raman signals after adding the anti-HER2 layer could be due to a screening effect of the latter.

The analysis of the surface roughness of 4-MBA SAMs and FSNP by AFM showed a slight change, which demonstrates that the film morphology was quasi-preserved. The surface roughness values (RMS_[Rq]_) were 4.83 ± 0.28 nm and 3.96 ± 0.83 nm, respectively. The process for calculating the surface roughness is reported in the work of Lara-Cruz et al. [40]. At this point, we consider that the FSNP prepared in this work accomplished all requirements to be employed as substrate for identification of breast cancer cells.

In the process of testing the affinity of breast cancer cells for FSNP, four cell lines with different HER2 expression (MCF-7, SKBR3, T47D and MDA-MB-231) were used. The following procedure was carried out: FSNPs were placed in duplicate for each cell line in 4-well multidish cell culture plates, an aliquot containing around 30,000 breast cancer cells (MCF-7, SKBR3, T47D, or MDA-MB-231) was dropped and incubated during 24 h in pseudo-physiological conditions (5% CO_2_ and 37 °C) and then fixed by ethanol dehydration train. Since our personal experience with formaldehyde is that it builds up a polymeric layer, which prevents the surface from being probed by an AFM tip, we performed fixation with ethanol dehydration train (ethanol-water solution) using solutions at 30%, 50%, 70%, 80%, 90%, 96% and 100% (*v*/*v*) for 3 min in each step.

The same process was carried out in parallel, using a clean Au(111) substrate, which supposedly lacks affinity for the cancer cell lines employed in this study. Both substrates were analyzed by AFM imaging that revealed morphological changes due to presumably different cell attachment. Nevertheless, to our surprise, the cell populations on both substrates were very similar for all cell lines, which could be attributed to a rather long time of cell incubation. As a further attempt to distinguish the difference between the four cell lines with differential HER2 expression, we studied the morphology and surface roughness of the cells attached to FSNPs.

**Figure 3:**
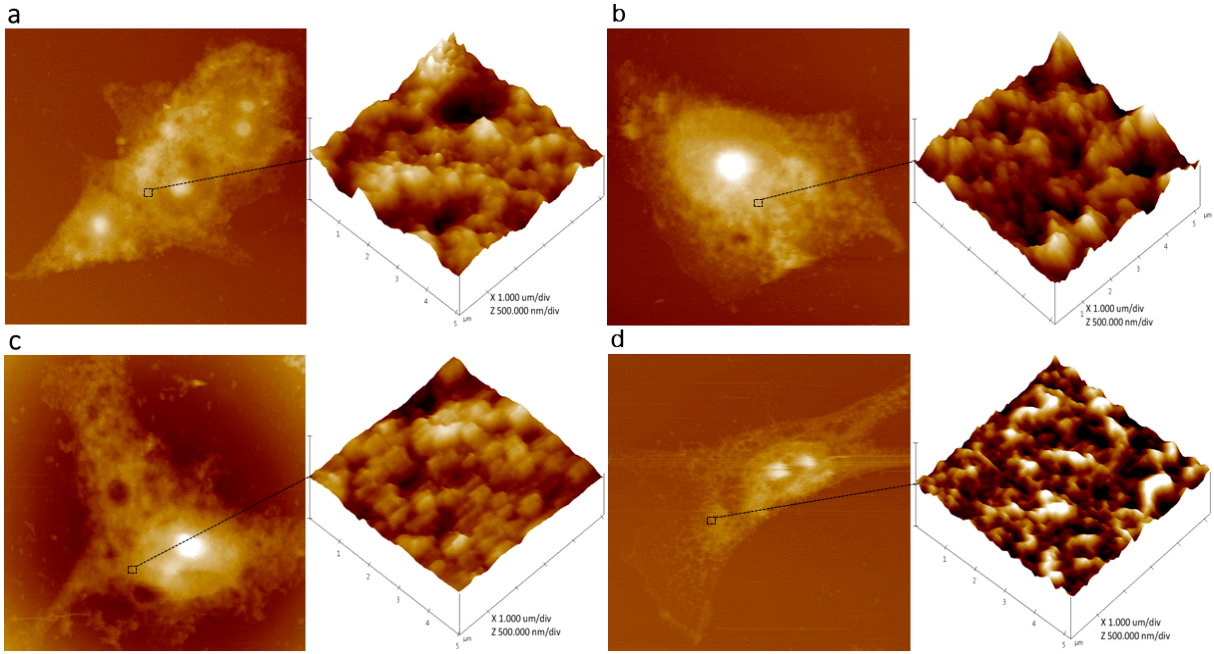
AFM image of (**a**) SKBR3 cells attached on a FSNP (image size: 55.5 µm × 55.5 µm, with z-scale: 0–3.0 µm); (**b**) MCF-7 cell attached on a FSNP (image size: 35.0 µm × 35.0 µm, with z-scale: 0–3.0 µm); (**c**) T47D cell attached on a FSNP (image size: 35.1 µm × 35.1 µm, with z-scale: 0–2.1 µm) and (**d**) MDA-MB-231 cell attached on a FSNP (image size: 50.0 µm × 50.0 µm, with z-scale: 0–3.0 µm). 3D images of the insert (5.0 µm × 5.0 µm) show a segment of the plasma membrane of each cell selected for surface roughness analysis.

AFM images shown in Figure 3a–d revealed different morphology for each cell line attached on FSNPs. Despite the imaged cells are of the same size, in many cases they possess two or more nuclei (typical for cancer cell lines) as well as cytoplasmic prolongations, which indicates a similar attachment to the surface. All AFM images are given in 2D top-view, however, for illustration a small segment (insert) is presented in 3D mode. Surface roughness of the parts obtained exclusively at the surface of the plasma membrane, was evaluated quantitatively and expressed as RMS_[Rq]_ factor. We calculated a paired t-test using the R statistical software [41] v3.4.1 in order to find the difference of means between the roughness of the plasmatic membranes adhered on a FSNP and on a flat Au(111) substrate. We only found significant differences (*p*-value < 0.05) in the surface roughness analysis of the plasma membranes of MCF-7 and T47D breast cancer cells. Note that RMS [Rq] and cellular height observed at AFM images are very low, probably due to the ethanol dehydration train.

Table 1 shows the results of the surface roughness analysis. In conclusion, we believe that AFM analysis did not satisfy our expectations and is not sufficiently good to discriminate different cell attachments or different cell lines with different HER2 expression.

**Figure 4:**
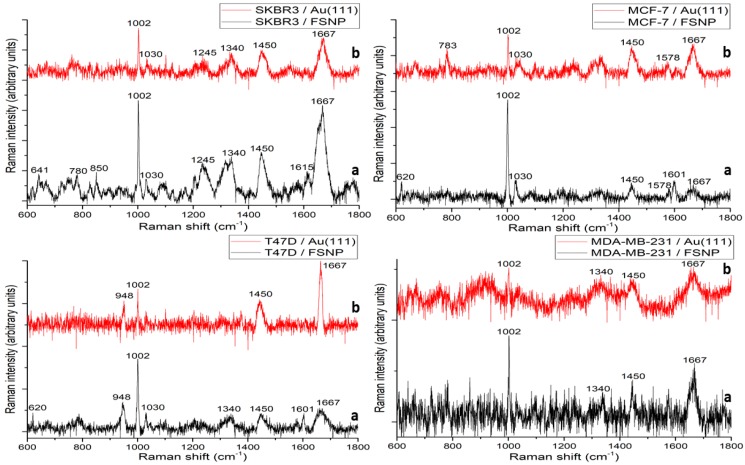
Raman spectra of four different breast cancer cell lines: SKBR3, MCF-7, T47D and MDA-MB-231 (**a**) attached on a FSNP and (**b**) attached on a flat substrate of Au(111).

Additionally, cellular samples were analyzed by SERS technique. Two types of substrates were used: a clean Au(111) substrate and our specially prepared FSNP with high affinity for HER2 expressed by the breast cancer cell lines analyzed. All Raman measurements were performed at least 30 times in the range of 600–1800 cm^−1^, where the main peaks were observed. In all spectra, the peaks at 1002 cm^−1^ (Phe), 1030 cm^−1^ (Phe), 1340 cm^−1^ (Amide III), 1450 cm^−1^ (CH_2_ and CH_3_ asymmetric) and 1667 cm^−1^ (Amide I) were found. Figure 4 shows the obtained SERS spectra of four different breast cancer cell lines (SKBR3, MCF-7, T47D and MDA-MB-231) attached on FSNP (a) and on a flat Au(111) substrate (b), respectively.

A considerable difference was obtained in the spectra between the two substrates used. All spectra were taken through the cells, preferentially on the nucleus part. Raman spectra obtained on FSNP have higher peak intensity, better peak definition and higher S/N ratio. We believe this improvement is due to SERS phenomena, despite the fact that our cell layer is several times thicker than the theoretically predicted length evanescent electromagnetic field for a nanostructured surface, which is confined to less than 30 nm. Therefore, using FSNP provides specific attachment of the breast cancer cells with differential HER2 expression and better Raman spectra, which is a great advantage in comparison with the plain gold substrate. All Raman spectra shown were baseline-corrected using the adaptive iteratively reweighted penalized least squares algorithm by Zhang et al. [42] and their assignments are in accordance with scientific literature [43,44,45,46] and are summarized in Table 2.

The most prominent peaks identified in the Raman spectra for all cell lines are at: 1002 cm^−1^, 1030 cm^−1^ attributed to the phenylalanine symmetric ring breathing and the phenylalanine C-H, respectively. The band at 1667 cm^−1^ is due to the amino acid Amide I vibration. According to the scientific literature, the Raman band at 1667 cm^−1^ is mainly attributed to protein Amide I probably from the cell membrane. Some Raman bands can show an overlap of vibrations and others can be Raman inactive. Therefore, using FSNP with high affinity and specificity for breast cancer cells will be of great advantage in the process of detection of such illness by SERS.

**Figure 5:**
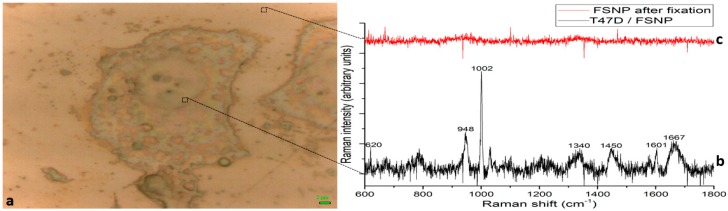
(**a**) Optical image of a T47D cell attached on a FSNP; (**b**) Raman spectrum of the T47D breast cancer cell attached on a FSNP and (**c**) Raman spectrum of the FSNP after cell fixation by an ethanol dehydration train. Scale bar: 2 μm.

The optical image in Figure 5a shows a T47D breast cancer cell attached on a FSNP (just as an example). This breast cancer cell line was incubated during 24 h on a FSNP under pseudo-physiological conditions (37 °C, 5% CO_2_) and then fixed with an ethanol dehydration train. Raman spectra were taken at two positions: one on the cell nucleus (Figure 5b) and another, on FSNP after cell fixation (Figure 5c). The Raman spectrum (Figure 5c) on the surface of FSNP does not present any features, which clearly shows that the above obtained spectra were originated from the cell material.

It means that the prepared FSNP could lead to the development of a biosensor for rapid, direct and highly sensitive identification of breast cancer cells.

**Figure 6:**
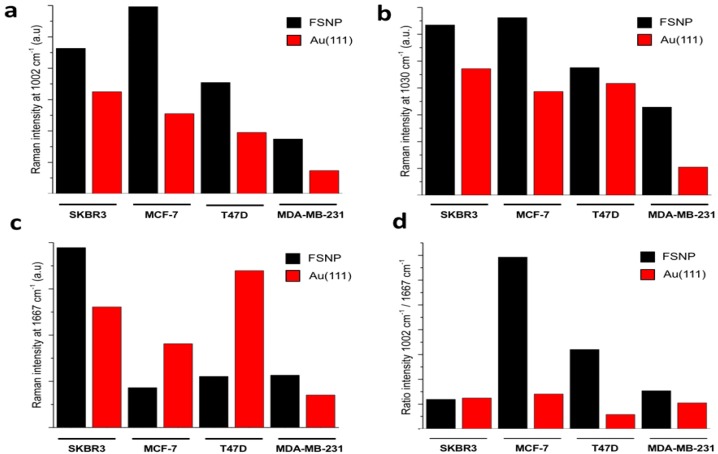
Raman intensities at (**a**) 1002 cm^−1^; (**b**) 1030 cm^−1^; (**c**) 1667 cm^−1^ and (**d**) the ratio 1002 cm^−1^/1667 cm^−1^ of the different breast cancer cell lines.

At this point, it is interesting to compare spectral peak characteristics between the four cell lines with differential HER2 expression. The obtained Raman peak positions are the same in all spectra, however the intensity ratio between the peaks within each cell line is different. Note that for comparisons, spectra with same S/N were used. The comparison was carried out between the ratio of the two prominent Raman peak intensities: 1002 cm^−1^ and 1667 cm^−1^. The ratios were determined at each spectrum independently and then compared as a function of HER2 expression (Figure 6a–d). The 1002 cm^−1^/1667 cm^−1^ ratio is proposed because these peaks are the most prominent in our Raman spectra. Furthermore, a direct relation between this ratio and the HER2 expression was seen in the different cell lines with the exception of SKBR3.

By means of this analysis, we found a tendency of the Raman intensity at 1002 cm^−1^, 1030 cm^−1^, 1667 cm^−1^ and 1002 cm^−1^/1667 cm^−1^ for the different breast cancer cell lines used in the present work. In Figure 6a–b, the Raman bands at 1002 cm^−1^ and 1030 cm^−1^ show a relationship similar to that found through CLSM. The Raman peaks located at 1002 cm^−1^ and 1030 cm^−1^ (associated with phenylalanine amino acid) for cell lines: SKBR3 and MCF-7 are of higher intensity than T47D and MDA-MB-231, which is in clear agreement with differential expression of HER2 surface receptor. These was observed for both surfaces: Au(111) and FSNP. Thus, using FSNP, the signal is very well defined and intense and has a higher signal-to-noise ratio. Therefore, FSNP is better option for SERS than the Au(111) plate. Based on the obtained results, we propose a new platform Figure 6c does not show any trend. We have evaluated Figure 6d that shows the ratio between the Raman intensity at 1002 cm^−1^ and 1667 cm^−1^, except for the data of SKBR3 cell line showing an unusual intensity at 1667 cm^−1^. We considered that it follows the same tendency mentioned before. The data presented here clearly show that SERS technique with a FSNP could be a powerful platform for cancer cell detection and identification of breast cancer cells with differential HER2 expression. A further development of this new platform includes its evaluation under physiological conditions.

## 4. Conclusions

Four breast cancer cell lines with different HER2 expression were studied by means of three different techniques: CLSM, AFM and SERS. The goal was to develop a platform for possible cancer cell identification and distinction between cells with different HER2 expression. Results obtained by CLSM show differences in the presence and distribution of HER2 on the cell surface membranes. HER2 expression from high to low was found to follow the order: SKBR3, MCF-7, T47D and MDA-MB-231, respectively. The obtained results are in agreement with the previous literature and knowledge.

AFM images obtained at nanometric level with high resolution show differences in surface morphology of the investigated cell lines but without significant changes in surface roughness to be used as a basis for development of a new platform. In order to use highly sensitive SERS, a new type of substrate was developed: functionalized silver nanostructured plate (FSNP) with a preadsorbed anti-HER2 (trastuzumab) layer, which provides a specific interaction between FSNP and HER2 expressed at the cancer cell surface. Indeed, our SERS results revealed that breast cancer cells show high affinity for the FSNP substrate, higher than for the bare gold samples. The SERS spectra of higher quality (higher S/N ratio and the excellent peak definition) were obtained with FSNPs. Among the different peaks identified in the spectra for different cell lines, peaks of higher intensity appear at: 1002 cm^−1^ and 1030 cm^−1^, associated with the phenylalanine amino acid.

The analysis of SEARS peaks shows that cell lines with higher HER2 expression (SKBR3 and MCF-7) exhibit higher Raman intensity at 1002 cm^−1^ than T47D and MDA-MB-231. These findings are in agreement with our results obtained by immunofluorescence, so there is some correlation between the two techniques. We can say that the most intense SERS signal is probably due to the coupling between trastuzumab and the silver nanostructured surface.

In general, the tendencies of the SERS effect with the FSNP and Au(111) plate are very similar; however, in the case of FSNP, the signal is very well defined and intense and has a higher signal-to-noise ratio. Therefore, we believe that the FSNP is better option for SERS than the Au(111) plate. Based on the obtained results, we propose a new platform capable of recognizing breast cancer cells with HER2 expression by using a FSNP with SERS technique. This platform could be used for cells with different HER2 expression levels and is mostly based on the identification of the most prominent Raman peak at 1002 cm^−1^.

## Figures and Tables

**Table 1:** Measurements of the surface roughness by AFM of the different breast cancer cell plasma membranes. The results are shown as: mean ± S.D.

Breast Cancer Cell Line	RMS_[Rq]_ (nm)
SKBR3/FSNP	48.9 ± 15.2
SKBR3/Au(111)	39.3 ± 7.2
MCF-7/FSNP	43.0 ± 6.8
MCF-7/Au(111)	58.9 ± 9.3
T47D/FSNP	50.1 ± 10.7
T47D/Au(111)	88.4 ± 27.8
MDA-MB-231/FSNP	40.2 ± 13.3
MDA-MB-231/Au(111)	43.4 ± 8.1

**Table 2:** Tentative Raman assignments. Abbreviations: (tw) twist, (U) uracil, (A) adenine, (G) guanine, (C) cytosine, (T) thymine, (br) breathing, (str) stretching, (bk) backbone, (sym) symmetric, (def) deformation, (p) protein.

Raman Shift (cm^−1^)	Tentative Assignment
1667	T, G, C, p: Amide I random coil
1615	Tyr, Trp, p: C=C
1601	Phe, Tyr, p: C=C
1578	A, G
1450	CH_2_ def
1335–1340	A, G, p: CH def
1245	Amide III ß-sheet
1030	C-H Phe
1002	Phe sym ring br
948	N-C_α_-C
850	Tyr ring br
780–783	U, T, C ring br; O-P-O str bk
641	C-C Tyr tw
620	C-C Phe tw
